# A novel TRPV4-specific agonist inhibits monocyte adhesion and atherosclerosis

**DOI:** 10.18632/oncotarget.9376

**Published:** 2016-05-14

**Authors:** Suowen Xu, Bin Liu, Meimei Yin, Marina Koroleva, Michael Mastrangelo, Sara Ture, Craig N. Morrell, David X. Zhang, Edward A. Fisher, Zheng Gen Jin

**Affiliations:** ^1^ Aab Cardiovascular Research Institute and Department of Medicine, University of Rochester School of Medicine and Dentistry, Rochester, NY, USA; ^2^ Department of Medicine, Medical College of Wisconsin, Milwaukee, WI, USA; ^3^ Department of Medicine, Division of Cardiology, and The Marc and Ruti Bell Program in Vascular Biology, New York University School of Medicine, New York, NY, USA

**Keywords:** AMPK, atherosclerosis, GSK1016790A, shear stress, TRPV4

## Abstract

TRPV4 ion channel mediates vascular mechanosensitivity and vasodilation. Here, we sought to explore whether non-mechanical activation of TRPV4 could limit vascular inflammation and atherosclerosis. We found that GSK1016790A, a potent and specific small-molecule agonist of TRPV4, induces the phosphorylation and activation of eNOS partially through the AMPK pathway. Moreover, GSK1016790A inhibited TNF-α-induced monocyte adhesion to human endothelial cells. Mice given GSK1016790A showed increased phosphorylation of eNOS and AMPK in the aorta and decreased leukocyte adhesion to TNF-α-inflamed endothelium. Importantly, oral administration of GSK1016790A reduced atherosclerotic plaque formation in ApoE deficient mice fed a Western-type diet. Together, the present study suggests that pharmacological activation of TRPV4 may serve as a potential therapeutic approach to treat atherosclerosis.

## INTRODUCTION

The vascular endothelium lining inner surface of blood vessels regulates vasodilation, which is compromised in the pathological states of many cardiovascular diseases such as atherosclerosis, hypertension, and stroke [1]. Atheroprotective laminar flow induces endothelium-dependent vascular relaxation partially through the release of the endothelium-derived nitric oxide (NO) [2, 3]. Recent studies have indicated that laminar flow promotes endothelial NO synthase (eNOS) activation followed by NO production through multiple mechanisms [4–6]. Although atherosclerosis can be treated by lipid-lowering statins, this protection remains incomplete and cardiovascular disease remains the leading cause of morbidity and mortality worldwide [7]. Therefore, laminar flow-mimetic compounds are, thus, of considerable interest for their potential therapeutic benefit in treating cardiovascular diseases.

Recently, a growing body of evidence has indicated that activation of the transient receptor potential ion channel vanilloid subtype 4 (TRPV4) plays an essential role in flow-mediated vasodilation in animal vascular beds and human coronary arterioles [8, 9]. Previous studies have demonstrated that TRPV4 is a thermo-[10, 11], osmo-[12], and mechano-sensitive [13, 14] ion channel, which can also be activated by pharmacological agonists. GSK1016790A is a recently discovered novel chemical entity that potently and selectively activates TRPV4 channel in a variety of recombinant and cellular assays [15, 16]. Over the past several years, GSK1016790A has been extensively used to study the pathophysiological role of TRPV4 in flow-mediated NO production and vascular function [14, 17]. More recently, it has been demonstrated that laminar flow- and TRPV4 agonist-induced vasodilation was impaired in aged rat mesenteric arteries, which was restored by lentivirus-mediated TRPV4 overexpression [18]. Laminar flow also sensitizes the response of TRPV4 to GSK1016790A by promoting the trafficking of TRPV4 from intracellular compartments to the plasma membrane [19]. However, it remains unknown whether pharmacological activation of TRPV4 by GSK1016790A can elicits laminar flow signaling events and prevents the development of atherosclerosis.

## RESULTS

### GSK1016790A stimulates eNOS activation in human endothelial cells

**Figure 1:**
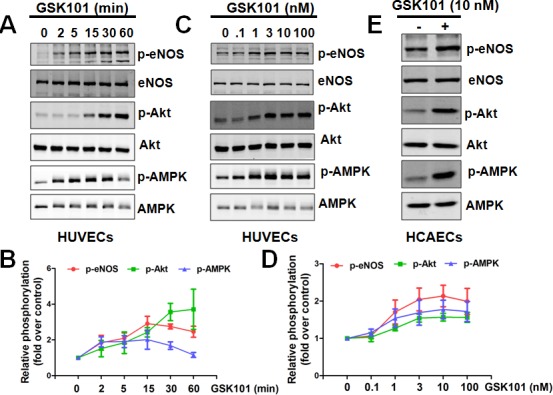
GSK1016790A activates eNOS signaling in human endothelial cells **A.-D.**, Time course (A-B) and dose-dependency (C-D) of GSK1016790A (GSK101) stimulation in HUVECs. HUVECs were treated with 10 nM GSK101 for indicated times or with indicated concentration of GSK101 for 15 min, then phosphorylation of eNOS, Akt, and AMPK were analyzed by Western blotting. Data were expressed as means ± SEM. **E**., HCAECs were treated with vehicle or 10 nM GSK101 for 15 min, then phosphorylation of eNOS, Akt, and AMPK were analyzed by Western blotting.

It is well established that laminar flow promotes NO production via eNOS phosphorylation (Ser1177) [20, 21]. To determine whether GSK1016790A can mimic flow to stimulate eNOS phosphorylation in cultured ECs, human umbilical vein ECs (HUVECs) were treated with GSK1016790A for up to 60 min. As shown in Figure 1A-1D, GSK1016790A significantly triggered the phosphorylation of eNOS, Akt and AMPKα, in a time-dependent and dose-dependent manner. As shown in Figure S1, we found that TRPV4 gene was specifically expressed in human endothelial cells but not monocytes and differentiated macrophages. We next determined whether GSK1016790A activates eNOS phosphorylation in human coronary artery EC (HCAECs). GSK1016790A also significantly induced eNOS phosphorylation, as well as the phosphorylation of Akt and AMPK in HCAECs (Figure 1E). Because eNOS can be phosphorylated at Thr495 that inhibits eNOS activity [22, 23], we also assessed the effect of GSK1016790A on eNOS phosphorylation at Thr495. We observed that eNOS was constitutively phosphorylated at Thr495 in HUVECs, and was rapidly dephosphorylated after GSK1016790A stimulation (Figure S2).

### GSK1016790A stimulates calcium- and TRPV4-dependent eNOS activation

**Figure 2:**
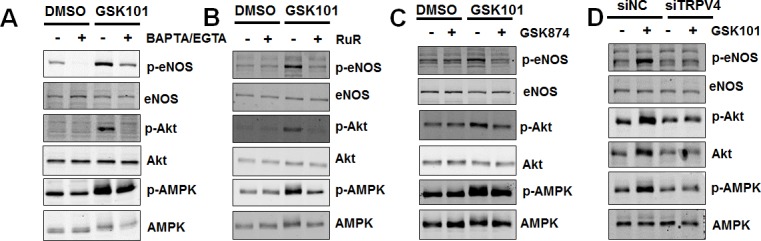
GSK1016790A-mediated signaling is calcium- and TRPV4-dependent **A.** HUVECs were pretreated with vehicle (0.1% DMSO), or calcium chelator BAPTA (3 μM) plus EGTA (500 μM) for 30 min, and then exposed to GSK1016790A (GSK101, 10 nM) for 15 min. **B.** HUVECs were pretreated with vehicle (H_2_O), or non-selective TRPV4 inhibitor Ruthenium Red (*RuR*; 5 μM) for 30 min, and then exposed to GSK101 (10 nM) for 15 min. **C.** HUVECs were pretreated with vehicle (0.1% DMSO), or TRPV4 specific antagonist GSK2193874 (GSK874, 100 nM) for 30 min, and then exposed to GSK101 (10 nM) for 15 min. **D.** HUVECs were transfected with 25 nM non-target control siRNA (siNC) or siRNA against TRPV4 (siTRPV4) for 48 h and then exposed to GSK101 (10 nM) for 15 min. Cell lysates were analyzed for Western blots using antibodies indicated.

Previous studies have shown that TRPV4 is critical for flow-dependent calcium entry and vasodilation [14, 24]. We next asked whether depletion of intracellular and extracellular calcium (by BAPTA/EGTA) or inhibition of calcium influx (by Ruthenium Red, RuR, a pan-TRPV4 antagonist) could inhibit GSK1016790A-mediated eNOS phosphorylation. We found that both BAPTA/EGTA and RuR restored eNOS-Ser1177 phosphorylation close to basal level (Figure 2A-2B). Laminar flow did not alter TRPV4 mRNA, or protein expression (Figure S3). However, depletion of TRPV4 by siRNA inhibited laminar flow-induced eNOS phosphorylation (Figure S4). In addition, pharmacological inhibition of TRPV4 activity by GSK2193874 [25] or transfection of HUVECs with human TRPV4 siRNA diminished GSK1016790A-induced eNOS phosphorylation at Ser1177 (Figure 2C-2D).

### GSK1016790A promotes eNOS activation partially through the CaMKK/AMPK pathway

**Figure 3:**
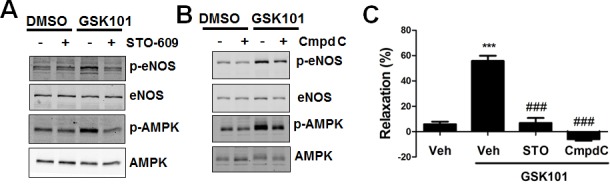
GSK1016790A activates eNOS partially through the CaMKK/AMPK pathway **A.** HUVECs were pretreated with vehicle (0.1% DMSO), or the CaMMK inhibitor STO-609 (1 μM) for 30 min, and then exposed to GSK1016790A (GSK101, 10 nM) for 15 min. **B.** HUVECs were pretreated with vehicle (0.1%DMSO), or the AMPK inhibitor Compound C (3 μM) for 30 min, and then exposed to GSK101 (10 nM) for 15 min. **C.** Aortas from C57BL/6J mice were pretreated with vehicle (0.1%DMSO), or STO-609 (1 μM), or Compound C (3 μM) for 30 min, then GSK101 (10 nM)-induced vasorelaxation of phenylephrine (PE, 1 μM)-precontracted aorta were determined. Values are mean ± SEM, ***p < 0.001 vs. vehicle control group. ^###^*p* < 0.001 vs. GSK101 group.

We next determined whether GSK1016790A activates AMPK activity, which is evidenced by phosphorylation of acetyl-CoA carboxylase (ACC), a substrate of AMPK [26]. We observed significant AMPK phosphorylation at Thr172, with attendant ACC phosphorylation at Ser79 after GSK1016790A stimulation (Figure S5). Calcium/calmodulin-dependent kinase kinase (CaMKK) is a calcium-sensitive AMPK upstream signaling molecule [27, 28]. Thus we tested the effect of the selective CaMKK inhibitor STO-609 on GSK1016790A-elicited AMPK and eNOS phosphorylation. We found that STO-609 almost abolished GSK1016790A-mediated AMPK and eNOS phosphorylation (Figure 3A). Similarly, we found that the AMPK inhibitor Compound C partially but significantly attenuated GSK1016790A-induced eNOS phosphorylation (Figure 3B). Moreover, STO-609 and Compound C reduced GSK1016790A-mediated vasorelaxation in aortic vessels (Figure 3C).

We next investigated the potential role of Akt in GSK1016790A-induced eNOS activation. We observed that LY294002 failed to block GSK1016790A-mediated eNOS phosphorylation at Ser1177 (Figure S6). Previous studies suggest that laminar flow can also phosphorylate eNOS at Ser1177 via the PKA-dependent pathway [29]. Our results showed that H-89, a selective inhibitor of PKA [29], failed to significantly inhibit GSK1016790A-dependent eNOS-Ser1177 phosphorylation, suggesting that PKA may not be involved in GSK1016790A-induced eNOS-Ser1177 phosphorylation (Figure S7). Collectively, our results suggest that GSK1016790A promotes eNOS activation partially through the CaMKK/AMPK-dependent pathway.

### GSK1016790A acutely activates eNOS and AMPK phosphorylation in mouse aorta

**Figure 4:**
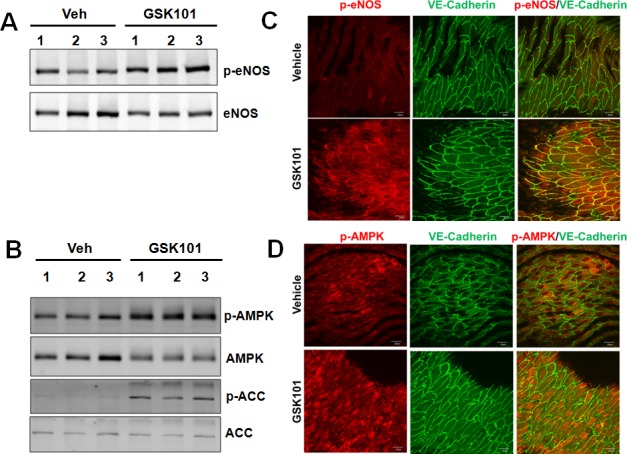
GSK1016790A treatment induces eNOS and AMPK phosphorylation in mouse aorta **A.-B.** Three-month old male C57BL/6J mice were injected (*i.p.*) with GSK1016790A (GSK101, 50 μg/kg body weight) or vehicle (*n* = 17 for detecting p-eNOS Ser1177; *n* = 6 for detecting p-AMPK and p-ACC). Thirty minutes after the treatment, mice were sacrificed and aortic tissues were harvested. Tissue lysates were analyzed by Western blots. **C.-D.** *En face* immunostaining of mouse aortic endothelium showing that GSK101 increased the phosphorylation of eNOS (p-eNOS Ser1177, red) and AMPK phosphorylation (p-AMPK Thr172, red) in aortic endothelium, and VE-cadherin (green) was used to label endothelial cells, DAPI was used to counterstain cell nuclei, bar = 30 μm.

To further validate and extend our *in vitro* results to more physiological settings, we tested whether treating normal C57BL/6J mice with GSK1016790A activates AMPK and eNOS phosphorylation in the mouse aorta. To this end, we injected (by *i.p.*) 12-week-old male mice with either GSK1016790A (50 μg/kg body weight) or vehicle. Western blot analysis revealed that a single *i.p.* injection of GSK1016790A resulted in increased phosphorylation of eNOS, AMPK and ACC in the aorta after 30 min (Figure 4A and 4B). *En face* immunofluorescent staining also revealed that GSK1016790A significantly promoted phosphorylation of eNOS at Ser1177 and AMPK at Thr172 in aortic endothelium (Figure 4C and 4D). Taken together, these results indicate that GSK1016790A acutely activates eNOS and AMPK in the intact aorta, highlighting the physiological significance of GSK1016790A for its potential to improve vascular function.

### GSK1016790A inhibits monocyte-endothelial cell adhesion in vitro and in vivo

**Figure 5:**
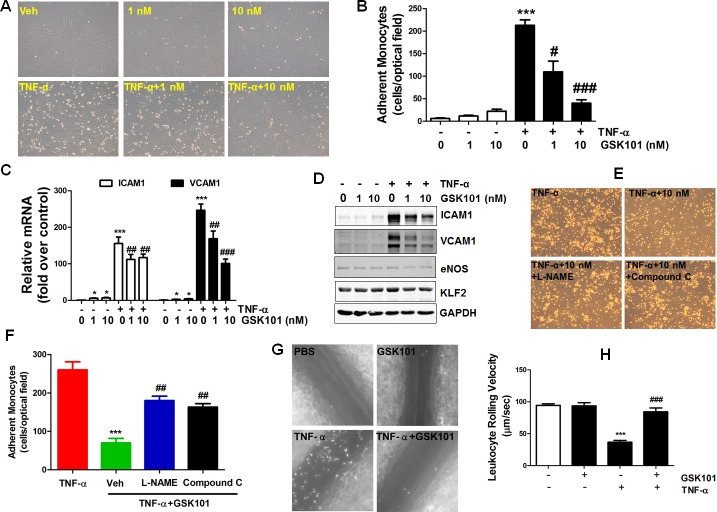
GSK1016790A attenuates monocyte adhesion to endothelial cells *in vitro* and *in vivo* **A.** HUVECs were pretreated with vehicle (DMSO), or GSK1016790A (GSK101, 1 nM and 10 nM) for 2 h, and then exposed to TNF-α (10 ng/ml) or vehicle (PBS) for an additional 6 h. Then, U937 monocytes was added for 30 min. Images were taken from representative optical fields showing endothelial cells (cobblestone shape) and adhering U937 monocytes (small, round cells) in the co-culture. **B.** Summary data for adherent monocytes described in A. **C.** and **D.** HUVECs were treated as described in A, then mRNA and protein expression of ICAM1 and VCAM1 were determined by qPCR and Western blotting, respectively. Values are mean ± SEM, *p < 0.05, ***p < 0.001 vs. vehicle control group. ^#^*p <* 0.05, ^##^*p* < 0.01, ^###^*p* < 0.001 vs. TNF-α group. **E**., HUVECs were pretreated with 100 μM eNOS inhibitor L-NAME for 30 min before treatment with GSK101 described in A. The images of U937 monocytic cells adherent to ECs were presented. **F.** Summary data for panel **E.** Values are mean ± SEM, ***p < 0.001 vs. TNF-α group. ^##^*p* < 0.01 vs. TNF-α +GSK101 group. **G.** Male C57BL/6 mice were pretreated with vehicle or GSK1016790A (10 mg/kg/d, by oral gavage) for 3 days, followed by vehicle or TNF-α injection (*i.p.*) for 4 h, intravital microscopy analysis was then performed to evaluate leukocyte adhesion to endothelium in the mesenteric microcirculation. Representative still images were shown for mice treated with vehicle (PBS), GSK101, TNF-α, or TNF-α plus GSK101. Summary data are provided in **H.** Values are mean ± SEM; *n* = 6-10; ***p < 0.001 versus PBS group, ^###^*p* < 0.001 versus TNF-α group.

eNOS-derived NO production prevents monocyte adhesion to ECs [30]. To determine whether eNOS activity enhancement by GSK1016790A improves EC function, the effect of GSK1016790A on tumor necrosis factor alpha (TNF-α)-induced monocyte adhesion to ECs was evaluated. We observed that TNF-α-induced monocyte adhesion was significantly reversed by the treatment of GSK1016790A (Figure 5A and 5B). The preventive effect of GSK1016790A against TNF-α-induced monocyte adhesion was due to decreased pro-inflammatory intracellular adhesion molecule-1 (ICAM1) and vascular cellular adhesion molecule-1 (VCAM1) mRNA and protein expression, but not related to a change in expression of anti-inflammatory molecules eNOS and krüppel-like Factor 2 (KLF2), (Figure 5C and 5D). Moreover, the inhibitory effect of GSK1016790A on monocyte adhesion was partially inhibited by L-NAME and Compound C (Figure 5E and 5F), suggesting the involvement of the AMPK/eNOS pathway.

To evaluate whether the anti-inflammatory effect of GSK1016790A in ECs translates to whole vessel, intravital microscopy analysis was performed to quantify leukocyte-ECs adhesion in mouse mesenteric microcirculation. Compared with vehicle control, TNF-α treatment significantly reduced leukocyte rolling velocity, and this effect was reversed by GSK1016790A pre-treatment (Figure 5G and 5H). Notably, GSK1016790A administration alone had no impact on leukocyte rolling velocity in vehicle-treated mice. These findings indicate that GSK1016790A inhibits inflammatory responses in the vasculature in vivo, by attenuating leukocyte adhesion to endothelium.

### GSK1016790A attenuates the development of atherosclerosis in ApoE−/− mice

**Figure 6:**
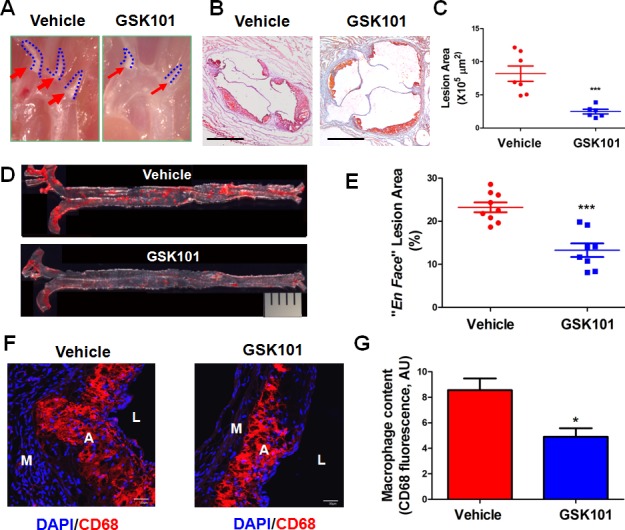
GSK1016790A attenuates the development of atherosclerotic lesions in ApoE−/− mice **A.** Representative images of atherosclerotic lesion formation in aortic arch of vehicle and GSK1016790A (GSK101)-treated ApoE^−/−^mice. **B.** Frozen sections from the aortic sinus of ApoE^−/−^ mice treated with vehicle or GSK101 for 4 weeks were stained with Oil Red O. The area of the Oil Red O-positive atherosclerotic lesions was quantified in **C.** Each symbol in the scatter plots represents the lesion area of one individual mouse. Values are mean ± SEM. **D.** Representative photographs from *en face* analysis of aortas from ApoE^−/−^ mice treated with vehicle or GSK101 for 4 weeks. The pictures presented were a composite of 4-6 images captured at different regions of the same aorta. The percentage of surface area of the Oil Red O-positive atherosclerotic lesions from the *en face* preparation relative to total luminal surface area of aorta was quantified in **E.** Each symbol in the scatter plots represents the lesion area percentage of one individual mouse. Values are mean ± SEM; ***p < 0.001 vs. vehicle group. **F.** GSK101 decreased macrophage content in atherosclerotic plaques. Aortic sinus from mice treated with vehicle or GSK101 were stained with rat anti-CD68 antibody to display macrophage content (Red), DAPI (Blue) was used to counterstain the nuclei, bar = 30 μm. L, lumen; A, atheroma; M, media. **G.** Quantification of CD68 positive staining of macrophages in panel F.

Endothelial dysfunction and leukocyte adhesion to inflamed endothelium are the hallmarks of early phase of atherosclerosis [30]. Thus, we explored the potential therapeutic efficacy of GSK1016790A in ApoE^−/−^ mice. Analyses of lipid profiles reveal that GSK1016790A did not alter total cholesterol (1406.0 ± 120.9 mg/dL *vs* 1361.0 ± 152.5 mg/dL, *n* = 5) or total triglyceride levels (64.0 ± 9.8 mg/dL *vs* 76.8 ± 14.5 mg/dL, *n* = 4-5). Gross observation of atherosclerotic lesions in the aortic arch showed that lesions were significantly reduced in GSK1016790A-treated mice (Figure 6A). In addition, the development of atherosclerotic plaques in aortic sinus was dramatically decreased in the mice treated with GSK101016790A (Figure 6B-6C). An *en face* preparation along the whole aorta also showed a stark contrast between the vehicle and GSK1016790A treated mice with regard to the percentage of Oil Red O-positive atherosclerotic plaques to total luminal surface area (Figure 6D-6E). Infiltrated monocytes differentiate to macrophages which uptake modified LDL to become foam cells, and thus causing atherosclerosis. We also observed decreased macrophage content in the aortic sinus of GSK1016790A-treated mice (Figure 6F-6G).

## DISCUSSION

TRPV4 is an important mechanosensing ion channel highly expressed in ECs that senses mechanical cues such as blood flow. Emerging evidence suggests that TRPV4 regulates vascular tone via NO generation [31]. Endothelial dysfunction, characterized by a decrease of NO bioavailability, is a hallmark of atherosclerosis [1]. Therefore, pharmacological activation of TRPV4 channel would confer atheroprotection. In the present study, we demonstrate that: 1) TRPV4 activation by GSK1016790A induces eNOS Ser1177 phosphorylation and activation in vascular ECs partially by activating the CaMKK/AMPK pathway; 2) GSK1016790A-elicted eNOS activation inhibits monocyte adhesion to ECs *in vitro* and leukocytes rolling *in vivo*; and 3) GSK1016790A attenuates the development of atherosclerosis in ApoE^−/−^ mice fed a Western diet. Our findings suggest that GSK1016790A suppresses vascular inflammation and has therapeutic potential for treating atherosclerosis.

The activation of eNOS is regulated by the coordinated phosphorylation of eNOS at multiple sites [32]. Specifically, phosphorylation of eNOS at Ser615, Ser633, and Ser1177 and dephosphorylation at Thr495 activates the enzyme [32]. In our study, we mainly focused on GSK1016790A-mediated eNOS phosphorylation at Ser1177, a major eNOS phosphorylation site contributed to eNOS activation [33, 34]. Because PKCα mediates acetylcholine-induced receptor-mediated TRPV4 activation in ECs [35] and PKCα stimulates eNOS phosphorylation on T495 [22, 36], we tested whether GSK1016790A decreases eNOS phosphorylation at Thr495 to further enhance eNOS activation. Our data suggest that GSK1016790A decreases eNOS phosphorylation at Thr495. Our results suggest that the combined effects of increased phosphorylation of eNOS at Ser1177 and dephosphorylation at Thr495 cooperatively enhance eNOS activation by GSK1016790A. One direction of our future studies could be to delineate the signaling mechanisms whereby GSK1016790A induces eNOS dephosphorylation at Thr495, such as the potential involvement of protein phosphatase 1 [22]. In addition, it remains to be investigated whether GSK1016790A affects other eNOS phosphorylation sites such as Ser615 and Ser633.

Multiple protein kinases are implicated in eNOS Ser1177 phosphorylation and subsequent activation, including Akt, AMPK, Ca^2+^/calmodulin-dependent protein kinase II (CaMKII), and PKA in a context-dependent manner [27, 32, 37–39]. For example, shear stress promotes eNOS phosphorylation at S1177 via the Akt [21, 33], AMPK [20] and PKA [29] dependent pathways and far infrared radiation increase eNOS phosphorylation at Ser1177 via a CaMKII-dependent manner [39]. We observed that the CaMKK inhibitor STO-609 abolished the effect of GSK1016790A on AMPK and eNOS phosphorylation, which suggests that CaMKK is the upstream kinase of AMPK and responsible for eNOS activation. Furthermore, the AMPK inhibitor Compound C partially inhibited GSK1016790A-mediated eNOS phosphorylation, suggesting that AMPK plays a partial role in GSK1016790A-stimulated eNOS phosphorylation. Using the PKA inhibitor H-89, we also excluded the possible involvement of a PKA [40] in GSK1016790A-mediated eNOS phosphorylation at Ser1177. This is different from TRPV1 channel activation by capsaicin, which activates PKA-dependent eNOS phosphorylation and vasorelaxation in spontaneously hypertensive rats [41].

Our results do not completely rule out the possibility that other kinases such as Akt [33, 34, 42, 43] and CaMKII [23, 39] may also play a role in GSK1016790A-mediated eNOS phosphorylation. Our data showed that pharmacological inhibition of PI3K/Akt did not significantly affect GSK1016790A-induced eNOS phosphorylation at S1177 at early time points. However, there are some literatures showing Akt functions downstream of AMPK [44, 45], so it is possible that Akt could be involved in the sustained activation of eNOS at S1177. Also, it remains unknown whether Akt is involved in GSK1016790A-induced eNOS phosphorylation at other activation sites, such as S615. Likewise, CaMKII may also play a role in GSK1016790A-mediated eNOS phosphorylation, a notion supported by the finding that pan-TRPV inhibition blocks far infrared radiation-induced CaMKII-dependent eNOS phosphorylation at Ser1177 [39]. The direct involvement of both kinases warrants further studies *in vitro* and *in vivo*.

It remains unknown regarding the precise role of TRPV4 in mouse and human atherosclerosis and whether endothelial function and atherosclerosis development will be exaggerated in TRPV4^−/−^; ApoE^−/−^ mice, or in ApoE^−/−^ mice treated with the TRPV4 inhibitor GSK2193874. Since atherosclerosis involves multiple cell types, thus systemic administration of GSK1016790A may potentially affect non-endothelial cells. In addition, we also recognize that additional long-term studies are needed to evaluate the pharmacokinetic properties and potential side effects (such as toxicity and vascular permeability) of GSK1016790A in animal model of atherosclerosis. Also, other potential beneficial downstream pathways (in addition to the activation of Akt, AMPK, and eNOS) activated by GSK1016790A remain to be investigated in future studies

In summary, the present study demonstrates that GSK1016790A, a specific and potent small-molecule activator of mechanosensitive ion channel TRPV4, induces eNOS phosphorylation and activation, and inhibits leukocyte adhesion to endothelium and the eventual development of atherosclerosis in mice. Our findings implicate that pharmacological activation of TRPV4 may serve as an effective and complementary strategy to the management of cardiovascular diseases.

## MATERIALS AND METHODS

### Chemicals and reagents

TRPV4 specific agonist GSK1016790A (GSK101, #G0798) and inhibitor GSK2193874 (GSK874, #5106) were obtained from Sigma-Aldrich (St. Louis, MO) and Tocris Bioscience (Bristol, UK), respectively. Compound C (AMPK inhibitor, #11967), STO-609 (calmodulin-dependent kinase kinase (CaMKK) inhibitor, #15325), Wortmannin (PI3K inhibitor, #10010591), LY294002 (Akt inhibitor, #70920), U0126 (ERK^1/2^ inhibitor, #70970), AICAR (#10010241), Forskolin (#11018), and Phorbol 12-myristate 13-acetate (PMA, #10008014) were obtained from Cayman Chemical (Ann Arbor, MI). Ruthenium Red (Ca^2+^ fluxes blocker, #557450), and H-89 (PKA inhibitor, #371963) were obtained from Calbiochem (San Diego, CA). Calcium chelator BAPTA/AM (#B-1205) was from Invitrogen (Grand Island, NY). Recombinant mouse TNF-α was from Roche (#11271156001, Indianapolis, IN). *N*_ω_-Nitro-L-arginine methyl ester hydrochloride (L-NAME, #N5751), Sodium nitroprusside (SNP, #PHR1423), Acetylcholine chloride (#A2661), (R)-(−)-Phenylephrine hydrochloride (PE, #P8155) and other chemicals were obtained from Sigma-Aldrich.

### Cell culture and laminar flow experiments

HUVECs were obtained from fresh umbilical cord veins from normal pregnancies with donors' informed consent. HUVECs were cultured in Medium 200 (#M-200-500, Thermo Fischer Scientific, Waltham, MA) containing 5% fetal bovine serum (FBS) and low serum growth supplement (LSGS) (#S-003-10, Thermo Fischer Scientific) [46]. HUVECs were collected in accordance with the University of Rochester human subjects review board procedures that prescribe to the Declaration of Helsinki. HCAECs (#300K-05A, Cell Applications Inc., San Diego, CA) were cultured in MesoEndo Cell Growth Media supplemented with Growth Supplement (#212K-500, Cell Applications Inc.) plus 5% FBS. HUVECs and HCAECs were subcultured with Trypsin/EDTA Solution (TE, #R001100, Thermo Fischer Scientific) and Trypsin Neutralizer Solution (TN, #R002100, Thermo Fischer Scientific). For steady laminar flow experiments, confluent HUVECs were cultured in the absence of serum for 24 h and exposed to laminar flow (shear stress of 12 dyne/cm^2^) in a cone and plate viscometer as we previously described [6]. Confluent HCAECs and HUVECs at passage 2 to 5 were used for all the experiments.

U937 cells (ATCC, Rockville, MD) were grown in RPMI 1640 Medium (Gibco) containing 10% FBS. THP-1 cells (gifted by Dr. Y. Cai), a pro-monocytic cell line, were cultured in RPMI 1640 supplemented with 10% FBS, 10 mM Hepes, 0.1 mM MEM non-essential amino acids, 1 mM sodium pyruvate, and 100 nM penicillin/streptomycin (all from Invitrogen). PMA (100 nM) was incubated with THP-1 cells for 48 h to induce monocyte differentiation into macrophages. Successful differentiation into macrophages was confirmed by cell morphology change, ICAM1 immunoblotting and oxidized LDL induced foam cell formation (by Oil Red O staining).

### siRNA oligonucleotide transfection

To knockdown TRPV4, HUVECs at greater than 80% confluence in 60-mm dishes were used for transfection [46]. In brief, lipofectamine 2000 or lipofectamine 3000 (6 μl; Invitrogen) was mixed with Opti-MEM (250 μl; Invitrogen), and then siGenome siRNA human TRPV4 (25 nM, D-004195-03, GE Dharmacon, Lafayette, CO) or non-target control siRNA (25 nM) diluted in 250 μl Opti-MEM was added to the solution, mixed gently, and incubated at room temperature for 20 min. A total of 0.5 ml of this mixture was added to HUVECs in 4 ml Opti-MEM and incubated for 4 h. Then medium was changed back to M200 complete medium and cells were treated after 48 h after transfection.

### RT-PCR and quantitative real-time PCR (qRT-PCR)

After treatment, total RNA was extracted using a QIAGEN RNeasy Mini kit (Qiagen). RNA concentration and purity were determined by Nanodrop2000 Spectrophotometer (Thermo Fischer Scientific). For reverse transcription, 0.5-1 μg of total RNA was converted into first strand complementary DNA (cDNA) in a 20 μl using a High-Capacity cDNA Reverse Transcription Kit (#4374966, Applied Biosystems, Foster City, CA) following the manufacturer's instructions. Regular RT-PCR was performed using 2X GoTaq Master Mix (#M7123, Promega). Reaction products were separated in 1% agarose gel and visualized with Image Lab 5.1 software (Bio-rad, Hercules, CA). Quantitative real-time PCR was then performed with a Bio-Rad iQ5 real-time PCR thermal cycler, using iQ SYBR Green Supermix (#1708886, Bio-Rad) for relative mRNA quantification. All primer sequences were listed in Table S1. The comparative cycle threshold (Ct) method (2^−ΔΔCt^) [47] was used to determine the relative mRNA expression of target genes after normalization to housekeeping gene GAPDH or β-actin.

### Western blot analysis

Western blot was performed using standard protocols [46]. In brief, cells were harvested in lysis buffer containing 20 mM Tris-HCl (pH 7.5), 150 mM NaCl, 1% Triton X-100, 1 mM EDTA, 1 mM EGTA, 2.5 mM sodium pyrophosphate, 1 mM β-Glycerolphosphate, 50 mM NaF, 1 mM Na_3_VO_4_ and supplemented with protease inhibitor cocktail (#P8340, Sigma-Aldrich). After centrifugation at 4°C (14, 000 rpm) for 20 min, protein extract supernatant was collected. The protein concentrations in the lysates were determined using the Bradford protein assay kit (#500-0006, Bio-rad) using a Beckman DU-800 spectrophotometer (Fullerton, CA). For Western blots, total cell lysates (10-20 μg) were separated by SDS-PAGE, transferred to nitrocellulose membrane (Pall, East Hills, NY) and were subsequently blocked in diluted (1:1, in PBS) Odyssey® blocking buffer (#927-40000, LI-COR Biosciences, Lincoln, NE) at room temperature for 1 h. Then the blots were incubated overnight at 4°C with 3%BSA-diluted primary antibodies (listed in Table S2). After being washed 3 times with 1 X Tris Buffered Saline with 0.1% Tween-20 (TBST), membranes were incubated with IRDye® 680RD Goat anti-Mouse IgG (H+L) or IRDye® 800CW Goat anti-Rabbit IgG (H + L) (1:10,000 dilution in 1XTBST; LI-COR) at room temperature for 30 min. Images were visualized by using an Odyssey Infrared Imaging System (LI-COR). Densitometric analysis of blots was performed with NIH Image J software (http://imagej.nih.gov/ij/) or Image Studio^Lite^ software (LI-COR).

### Vascular reactivity experiments

Mouse thoracic aorta from three-month-old C57BL/6J mice were dissected and mounted in a four-chamber Multi-wire Myograph System (DMT-610M, Arhus, Denmark) [48, 49]. Vascular segments were dissected free of loose connective and peri-aortic adipose tissue under dissecting microscope (Olympus SZX7), and cut into rings of equal length (2 mm). Artery segments were maintained at 37°C in Krebs physiological saline solution (PSS) of the following composition (in mM): 118.3 NaCl, 4.7 KCl, 2.5 CaCl_2_, 1.2 MgSO_4_, 25 NaHCO_3_, 1.2 KH_2_PO_4_, and 5.5 D-Glucose. PSS was pre-warmed at 37°C and saturated with air balanced-5% CO_2_ to maintain a pH of 7.4. Arteries were subject to a wake-up protocol by stimulation two times with a 60 mM-K^+^ Krebs solution (K-PSS), in which NaCl was substituted with KCl of equal molar concentration, for 3 min each at 10-min intervals. Vessels were then constricted to 50-75% of maximum KCl responses with PE (1 μM). After the contraction reached plateau state, relaxation responses to the selective TRPV4 agonists GSK1016790A (10 nM) were determined in the absence or presence of following inhibitors: STO-609 (1 μg/ml) and Compound C (3 μM). The endothelium was considered intact if acetylcholine (10 μM) caused >80% relaxation of phenylephrine (PE)-pre-contracted arteries. Vasodilatory responses were expressed as percent relaxation relative to PE-induced vascular tone, with 100% representing full relaxation to basal tension. Force was recorded via a PowerLab 4/30 system (AD Instruments Ltd., UK) and analyzed using LabChart 7.0 Acquisition System (AD Instruments Ltd., UK).

### Monocytes-endothelial cells adhesion assay

To evaluate monocyte adhesion to ECs [6], confluent HUVECs monolayers were pretreated with GSK101 (0 nM, 1 nM, 10 nM) for 30 min followed by treatment with 10 ng/ml TNF-α for 6 h. To examine whether the effect of GSK101 were mediated through eNOS and AMPK, HUVECs were pretreated with vehicle, or 100 μM L-NAME (in PBS, an eNOS inhibitor) or 3 μm Compound C for 30 min before exposure to 10 nM GSK101 for 30 min, and then exposed to TNF-α (10 ng/ml) for 6 h. After treatment, the media were then removed, and U937 cells were added to dishes and incubated for 30 min at 37°C. Non-adhering cells were removed by gentle washing 3 times with serum-free medium. The adherent cells were immediately counted in each well. Phase-contrast microphotographs of the cells in plates were taken with Zeiss Axiovert 40C microscope (magnification: ×10; numeric aperture: 0.25; Carl Zeiss) using a Canon A640 digital camera (Canon USA Inc).

### Mice and drug treatment

To assess the potential therapeutic efficacy of GSK1016790A on the development of atherosclerosis, 8-week-old ApoE^−/−^ mice (The Jackson Laboratory, Bar Harbor, ME) were fed a Western-type diet (#TD.88137, Harlan Teklad) for 8 weeks. After 8 weeks, the mice were continued on Western diet for an additional 4 weeks and orally administered (by oral gavage) once per day during this period with GSK1016790A (10 mg/kg/d) which was dissolved in 2% carboxymethyl cellulose sodium (CMC-Na, Sigma) [50, 51]. Atherosclerotic lesion areas in *en face* aorta and aortic sinus were analyzed as we previously described [47, 52]. Blood serum was prepared for biochemical analysis of lipid profile at University of Rochester Labs Clinics. To determine whether GSK1016790A can stimulate the phosphorylation of eNOS, AMPK and ACC in mouse aorta, 12-week-old male C57BL/6J mice (The Jackson Laboratory) were acutely injected with GSK1016790A at 50 μg/kg body weight intraperitoneally (*i.p.*) (dissolved in 5% DMSO/30%PEG200 in saline). Control animals received the same amount of vehicle injection. After 30 min, mice were sacrificed, and the whole aortas were subject to *en face* staining or dissected in chilled PBS and snap frozen in liquid nitrogen. Whole aortas were homogenized with Biospec Tissue Tearor and tissue lysate are subject to Western blot analysis. All animal studies were approved by the Institutional Animal Care and Use Committee of the University of Rochester Medical Center.

### Immunofluorescent staining

*En face* staining was performed as previously described [46]. After injection with GSK1016790A for 30 min (by *i.p.*), mice were anesthetized with ketamine/xylazine cocktail (0.13/0.0088 mg/g body weight). Then, the arterial tree was perfused with saline containing 40 USPU/ml heparin from left ventricle for 5 min, followed by perfusion of pre-chilled 4% paraformaldehyde in PBS (pH 7.4) for 10 min. Subsequently, after adipose tissues were removed, the whole aorta was dissected from thoracic aorta to the heart, cut open longitudinally, permeabilized with 0.1% Triton X-100 in PBS for 10 min and blocked with 10% normal goat serum (Invitrogen) in Tris-buffered saline (TBS) containing 2.5% Tween-20 for 1 h at room temperature. Next, aortas were incubated with rat anti-VE-Cadherin (also known as CD144, 1: 100, BD Bioscience, #555289), mouse anti-p-eNOS (Ser1177) antibodies or rabbit anti-p-AMPK (Thr172) antibodies in the antibody dilution buffer (3%BSA+TBS-2.5% Tween-20) overnight at 4oC. After rinsing with washing solution (TBS containing 2.5% Tween-20) 5 min for 3 times, aortic segments were incubated with Alexa Fluor 488 conjugate goat anti-rat and Alexa Fluor 546 conjugate goat anti-mouse secondary antibodies (1:1000 dilution) for 1 h at room temperature. Finally, after another 3 rinses in the washing solution, aortic specimens were gently placed on a glass slide with the luminal side up (under dissection microscope), mounted in the ProLong Gold-antifade Mounting Media (Invitrogen). For CD68 immunostaining, the aortic sinus were incubated with a rat anti mouse CD68 antibody (1:100, #MCA1957GA, AbD Serotec, Raleigh, NC) overnight at 4oC, and stained as mentioned above. Slides of aortic segments or aortic sinus were examined by a laser-scanning confocal microscope (FV-1000 mounted on IX81, Olympus) with UPlanFL N 60x Oil lens. Fluorescence expressed in arbitrary units (AU) was quantified using the Image J software.

### Intravital microscopy for assessing leukocyte-endothelial adhesion

Leukocyte-endothelial cell adhesion was evaluated using intravital microscopy as described previously [53, 54], with slight modifications. Briefly, 4-week-old male C57BL/6J mice (The Jackson Laboratory) were administrated by oral gavage with vehicle or GSK101016790A (10 mg/kg body weight) daily for 3 days, and on the following day, they received an intraperitoneal (*i.p.*) injection of vehicle (PBS) or murine recombinant tumor necrosis factor α (TNF-α) (#315-01A, PeproTech, Rocky Hill, NJ, 0.5 μg/mice). At 4 h after vehicle or TNF-α treatment, the mice were prepared for intravital microscopy. Mice were anesthetized with a cocktail of ketamine/xylazine (80:12 mg/kg) and saline (10 ml/kg) via *i.m.* injection. Endogenous leukocytes were fluorescence labeled by injection of the mice with Rhodamine-6G (100 μl of 0.05% solution given by optic vascular plexus), and the mesentery was exposed for the observation and recording of images of leukocyte adhesion and rolling using a Nikon ECLIPSE Ti inverted fluorescent intravital microscope (Nikon Americas. Melville, NY). 5-6 vessels per mouse were imaged, with each vessel recorded for at least one minute. The velocity of leukocyte rolling was calculated and images were processed using the Image Pro-Plus 6.0 software (Media Cybernetics, Rockville, MD).

### Morphometric analysis of atherosclerotic lesions

For lesion formation in aortic sinus, saline-perfused hearts were fixed in 4% paraformaldehyde/PBS overnight (4 oC) followed by dehydration in 30% sucrose overnight at 4 oC. Fixed aortic roots were frozen in OCT embedding medium (Sakura Finetek). Five serial 8-μm sections were cut at 20-μm intervals from the appearance of three aortic valves, and the sections were stained with 0.22 μm-filtered pre-warmed 0.3% Oil Red O working solution (#S1849, Poly Scientific, Bay Shore, NY) for 1 min, counterstained with hematoxylin (Sigma-Aldrich), and mounted with glycerol gelatin aqueous mounting medium (#GG1, Sigma-Aldrich). For quantitative analysis of atherosclerosis, the total lesion area of each mouse was quantitatively analyzed with the NIH Image J Software as previously described [47].

For lesion formation in *en face* aorta [51], Mice were sacrificed and the aortic tree was perfused with ice-cold phosphate-buffered saline (PBS) followed by 4% paraformaldehyde (PFA, pH 7.4); aortas spanning brachiocephalic artery to iliac artery bifurcation were excised and opened longitudinally. After removing the peripheral fat and adventitial tissue under a dissecting microscope, aortas were fixed overnight with 4% PFA, rinsed with PBS for 10 min, and briefly rinsed in 60% isopropanol (5 sec); stained with filtered pre-warmed 0.3% Oil Red O working solution for 10 min with gentle stirring on VWR rocking platform; and destained for 5 sec in 60% isopropanol followed by three washes with PBS; and finally laid out on Superfrost™ Plus Microscope Slides (Fischer Scientific) for *en face* presentation. The images of the aortas were captured (against a dark background) with ProgRes Speed XT^core^5 CCD camera (JENOPTIK AG, Germany) mounted on a microscope (Leica S8AP0, Germany). The pictures presented were a composite of 4-6 images captured at different regions of the same aorta. Percentage of Oil Red O-positive stained area in relation to total luminal surface area was quantified using computer-assisted morphometry (NIH ImageJ software version 1.48) [50].

### Statistics

Data were expressed as means ± SEM from three to six experiments unless indicated otherwise. Statistical analysis was performed using GraphPad Prism software 5.0 (GraphPad, La Jolla, CA). Student's *t* test and one-way analysis of variance (ANOVA) with *post hoc* Bonferroni tests were used for comparisons between two groups and multiple comparisons, respectively. A corrected *p* value less that 0.05 was considered statistically significant.

## SUPPLEMENTAL MATERIALS FIGURES AND TABLES


