# Supplementary material for: A novel TRPV4-specific agonist inhibits monocyte adhesion and atherosclerosis
Source: Oncotarget. 2016 May 14;7(25):37622–35. doi: 10.18632/oncotarget.9376 (PMC5122337; doi:10.18632/oncotarget.9376)
Supplement: Supplementary file 1 [file oncotarget-07-37622-s001.pdf]

A novel TRPV4-specific agonist inhibits monocyte adhesion and atherosclerosis

SUPPLEMENTAL MATERIALS

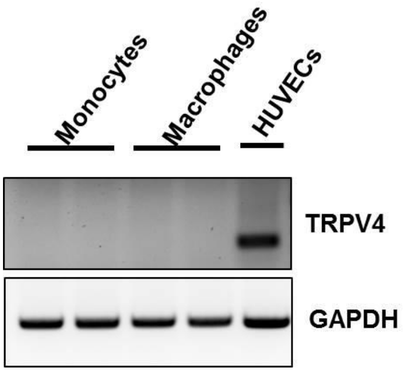

Figure S1. TRPV4 is specifically expressed in endothelial cells, but not in monocytes/macrophages. Human THP-1 monocytic cells were incubated with vehicle (0.1% DMSO) or 100 nM PMA for 48 h to induce differentiation into macrophages, then total RNA was isolated for RT-PCR, GAPDH was used as the loading control, RNA isolated from HUVECs was used as the positive control.

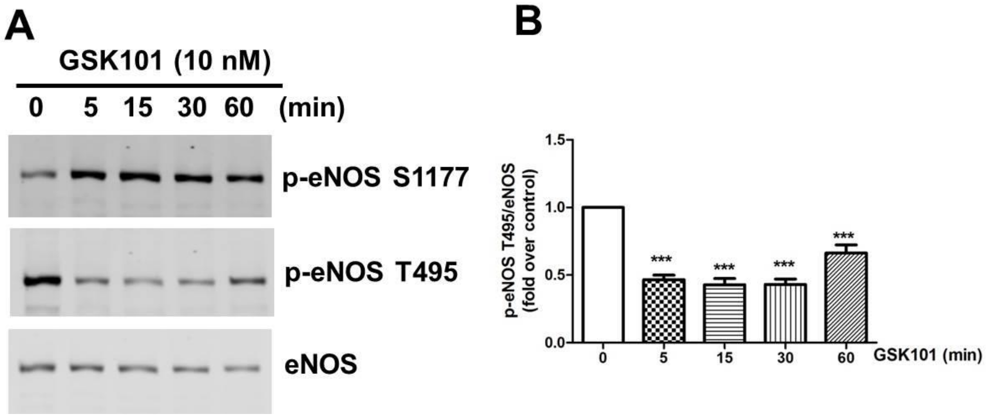

Figure S2. GSK1016790A promotes the phosphorylation of eNOS at S1177, while decreases the phosphorylation of eNOS at T495.

A, HUVECs were treated with 10 nM GSK1016790A (GSK101) for indicated times, then phosphorylation of eNOS at T495 and S1177 were analyzed by Western blotting. Total eNOS from the same stripped membrane was used as loading control.

B, Densitometric analysis of A. Data were expressed as means ± SEM, ****p*<0.001 vs control (0 min).

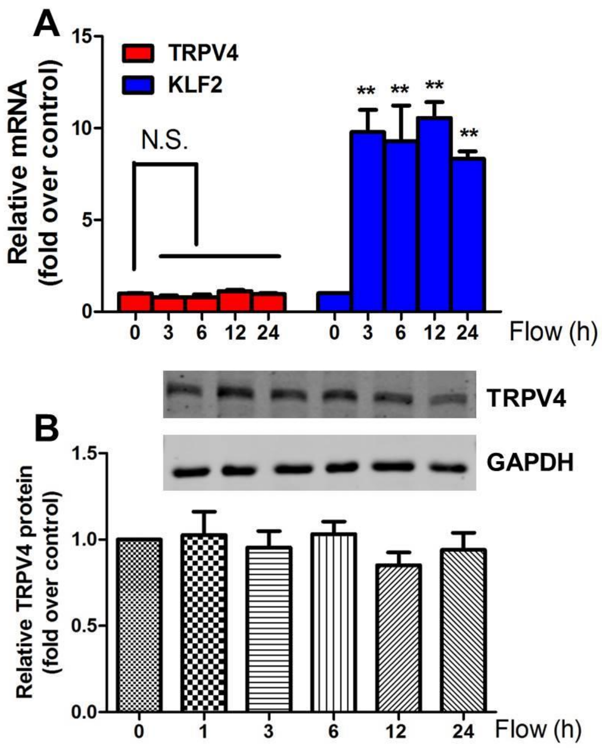

Figure S3. Laminar flow does not affect TRPV4 mRNA or protein expression

A, HUVECs were subjected to laminar flow for indicated time, then RNA was isolated for real time PCR analysis to examine TRPV4 gene expression. Laminar flow induced flow-responsive KLF2 gene upregulation from 3 h to 24 h, without affecting TRPV4 gene expression, **p<0.01 vs control. N.S. denotes non-significant difference.

B, HUVECs were treated as described in A, and whole cell lysates were collected for western blot analysis to detect TRPV4 protein expression.

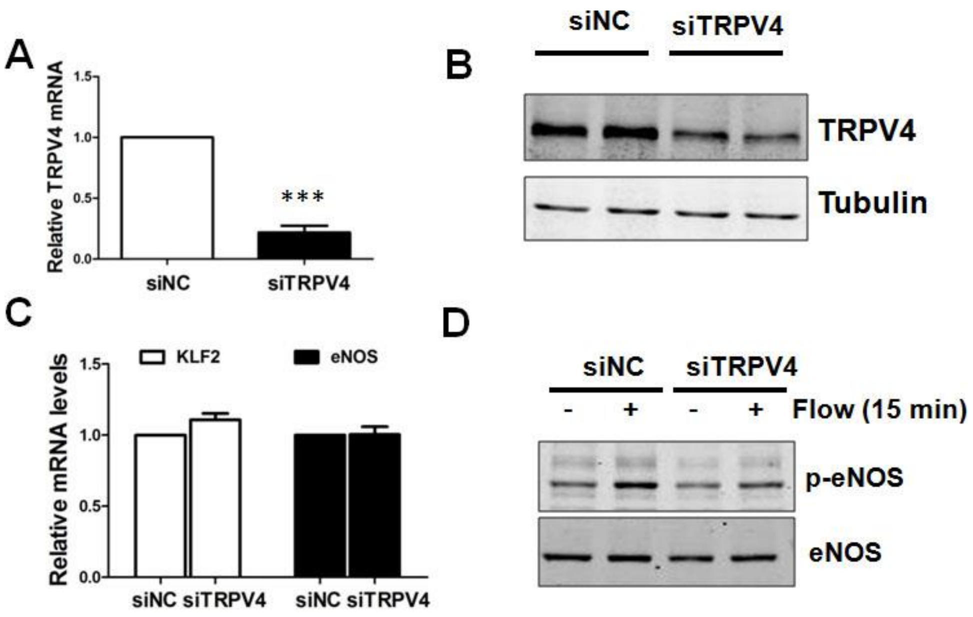

Figure S4. TRPV4 depletion inhibits flow induced eNOS phosphorylation but does not affect eNOS and KLF2 expression.

A-B, Silencing efficiency of TRPV4 siRNA. Knockdown of human TRPV4 was performed using TRPV4 siRNA (siTRPV4). A non-targeting siRNA Control (siNC) was used as a control. HUVECs were transfected with siNC (25 nM) or siTRPV4 siRNA (25 nM) with Lipofectamine 2000 (Invitrogen) for 4 h according to the manufacturer's instructions. Cells were cultured for an additional 48 h in complete media at 37°C, and mRNA and proteins were extracted for real time PCR (Panel **A**, ***p<0.001 vs. siNC) and Western blot (Panel **B**) analysis, respectively.

C, TRPV4 siRNA does not affect KLF2 and eNOS gene expression.

D, TRPV4 siRNA inhibits flow induced eNOS phosphorylation (Ser1177). HUVECs were treated as described in A, then subject to laminar flow for 15 min. Whole cell lysates were collected and subjected to Western blot using antibodies as indicated.

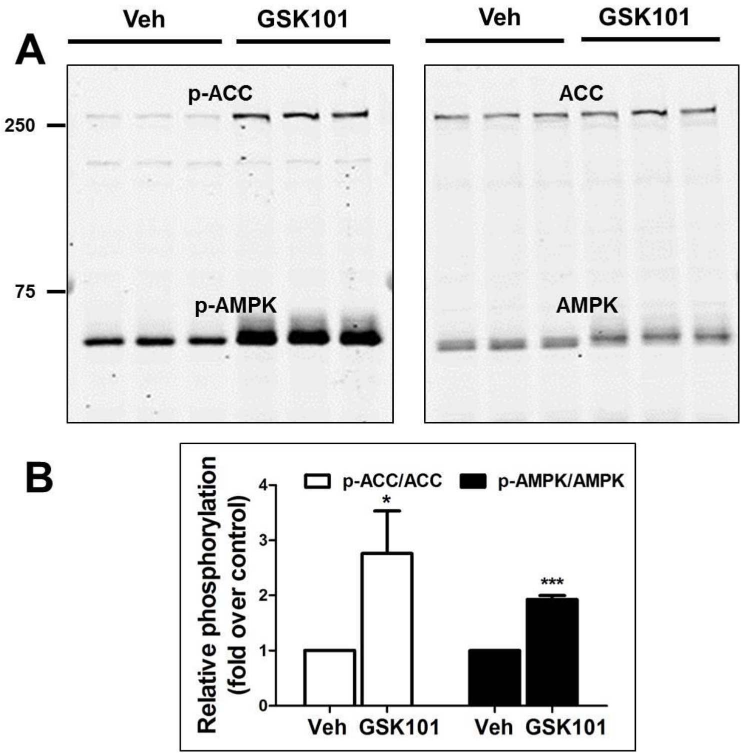

Figure S5. GSK1016790A stimulates the phosphorylation of acetyl-CoA carboxylase (ACC), a direct AMPK substrate.

A, GSK1016790A (GSK101) increases AMPK activation. HUVECs were treated with vehicle (0.1% DMSO) or 10 nM GSK101 for 15 min. Whole cell lysates of cultured HUVECs were probed by Western blot with antibodies against p-ACC, ACC, p-AMPK, and AMPK. Notably, after treatment with GSK101, the bands of p-AMPK and total AMPK display clear band shift.

B, Quantification of panel A, *$p<0.05$, ***$p<0.001$ vs vehicle.

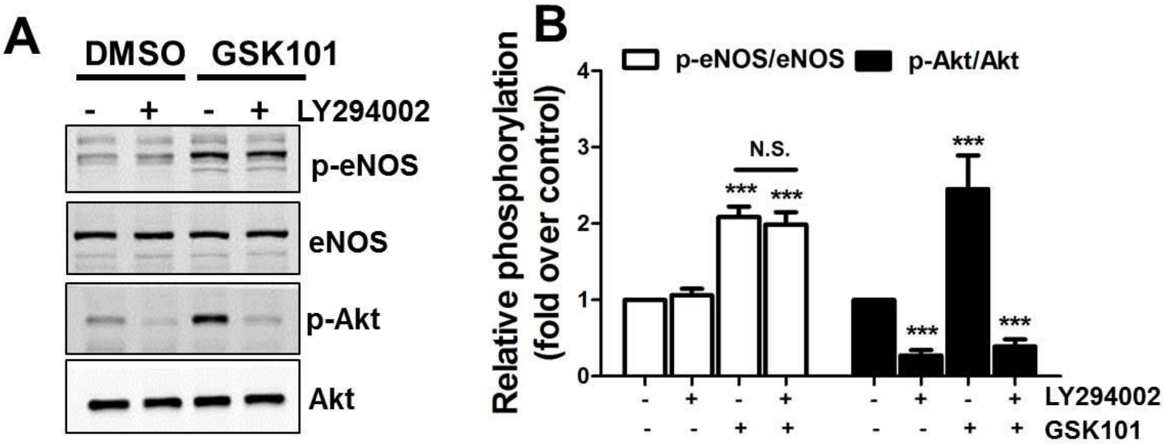

Figure S6. PI3K/Akt inhibitor LY294002 does not significantly block GSK1016790A-mediated eNOS phosphorylation.

A, HUVECs were treated with vehicle (0.1% DMSO) or the LY294002 (10 μM) for 30 min before treatment with 10 nM GSK1016790A (GSK101) for 15 min. Western blot was performed with antibodies against p-eNOS (S1177), eNOS, p-Akt and Akt.

B, Densitometric analysis of A. ****p*<0.001 vs vehicle control. N.S. represents no significance detected.

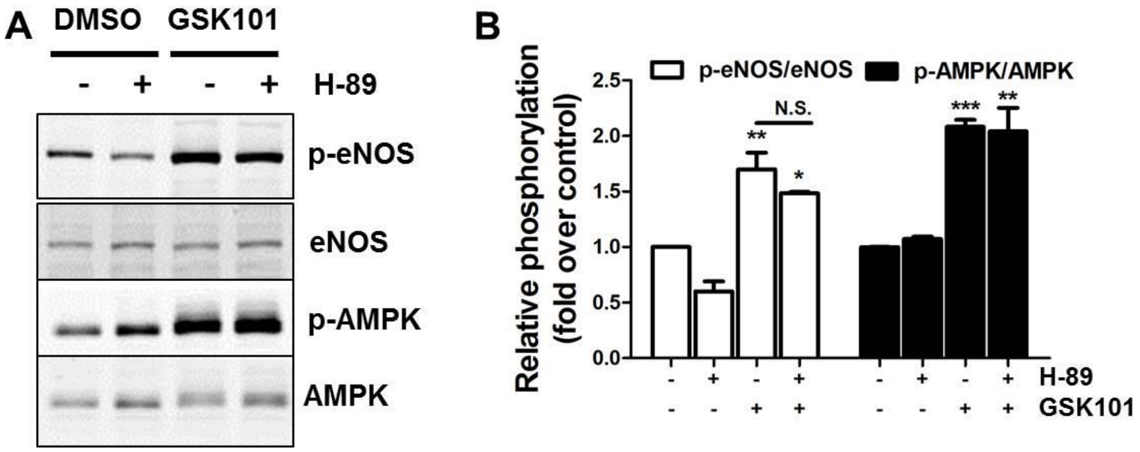

Figure S7. PKA inhibitor H-89 does not significantly block GSK1016790A-mediated eNOS phosphorylation.

A, HUVECs were treated with vehicle (0.1% DMSO) or the PKA inhibitor H-89 (1 μM) for 30 min before treatment with 10 nM GSK1016790A (GSK101) for 15 min. H-89 inhibits Forskolin induced PKA-dependent eNOS phosphorylation (data not shown) but has no significant effect on GSK101-mediated eNOS phosphorylation. Western blot was performed with antibodies against p-eNOS (S1177), eNOS, p-AMPK and AMPK.

B, Densitometric analysis of A. **p*<0.05, ***p*<0.01, ****p*<0.001 vs vehicle control. N.S. represents no significance detected.

Table S1. Primers sets for PCR

Real time PCR

QuantiTect Primer assays for human KLF2 (Hs_KLF2_1_SG, # QT00204729), eNOS (Hs_NOS3_1_SG, #QT00089033) and β-actin (Hs_ACTB_1_SG, # QT00095431) are obtained from QIAGEN.

Gene name	Sequence (5'-3')
**TRPV4**	Forward: CTGTTTGACTACGGCACCTATC<br>Reverse: CGGCTGCTTCTCTATGATCTTC
**ICAM1**	Forward: GGCCGGCCAGCTTATACAC<br>Reverse: TAGACACTTGAGCTCGGGCA
**VCAM1**	Forward: TCAGATTGGAGACTCAGTCATGT<br>Reverse: ACTCCTCACCTTCCCGCTC

RT-PCR

Gene name	Sequence (5'-3')	Amplicon
**TRPV4**	Forward: CTGTTTGACTACGGCACCTATC<br>Reverse: CTCCTCATCAGTTAGGCGTTTC	234 bp
**GAPDH**	Forward: GATTCCACCCATGGCAAATTC<br>Reverse: GTCATGAGTCCTTCCACGATAC	380 bp

Table S2. Sources of antibodies used in this study

Antibodies	Supplier, Cat. No.	Dilution
**TRPV4**	Alomone Labs, #ACC-034	1: 300
**eNOS**	BD, #610297	1: 1, 000
**p-eNOS (Ser-1177)**	Cell Signaling, #9571	1: 1, 000
**p-eNOS (Ser-1177)**	BD, #612393	1: 1, 000
**p-eNOS (Thr495)**	BD, #612706	1: 1, 000
**AMPKα**	Cell Signaling, #5831	1: 1, 000
**p-AMPKα (Thr-172)**	Cell Signaling, #2535	1: 1, 000
**ERK**	Cell Signaling, #9107	1: 1, 000
**p-ERK (Thr-202/Tyr-204)**	Cell Signaling, #4370	1: 1, 000
**Akt**	Cell Signaling, #4691	1: 1, 000
**p-Akt (Ser-473)**	Cell Signaling, #4060	1: 1, 000
**p-ACC**	Cell Signaling, #11818	1: 1, 000
**ACC**	Cell Signaling, #3676	1: 1, 000
**ICAM1**	Santa Cruz, #sc-8439	1: 1, 000
**VCAM1**	Santa Cruz, #sc-1504	1: 1, 000
**α-Tubulin**	Sigma, #T5168	1: 10, 000
**GAPDH**	EMD Millipore, #AB2302, #MAB374	1: 5, 000
**KLF2**	Abcam, #Ab189541	1: 1, 000
